# Forest maturity has a stronger influence on the prevalence of spider monkeys than howler monkeys in an anthropogenically impacted rainforest landscape

**DOI:** 10.1007/s10329-022-00980-8

**Published:** 2022-02-26

**Authors:** A. Shedden, J. C. Dunn, R. Martínez-Mota, J. Cristóbal-Azkárate, P. K. Gillingham, C. MacSwiney-González, A. C. Newton, E. Rodríguez-Luna, A. H. Korstjens

**Affiliations:** 1 Faculty of Science and Technology, Bournemouth University, Talbot Campus, Fern Barrow, Poole, BH12 5BB Dorset UK; 2 Behavioural Ecology Group, Anglia Ruskin University, Cambridge Campus, East Road, Cambridge, CB1 1PT UK; 3 Biological Anthropology, University of Cambridge, Cambridge, CB2 3QG UK; 4 Department of Cognitive Biology, University of Vienna, Althanstrasse 14, 1090 Vienna, Austria; 5 Centro de Investigaciones Tropicales, José María Morelos #44, Zona Centro, C.P. 91000 Xalapa, Veracruz México; 6 Department of Basic Psychological Processes and Their Development, University of the Basque Country, 20018 Donostia-San Sebastián, Spain

**Keywords:** Endangered primates, *Alouatta*, *Ateles*, Primate conservation, Habitat loss

## Abstract

The transformation and depletion of primary forest over the past few decades have placed almost half of the world’s primate species under the threat of extinction. Developing any successful conservation program for primates requires distribution and demography data, as well as an understanding of the relationships between these factors and their habitat. Between March and June 2010 and 2011 we collected data on the presence and demographic parameters of howler and spider monkeys by carrying out surveys, and validated our findings using local knowledge. We then examined the relationship between forest type and the presence of these primates at 54 sites in the northern area of the Selva Zoque Corridor, Mexico. We detected 86 spider monkey groups across 31 plots and censused 391 individuals (mean ± SD = 5.9 ± 3.0 individuals per sub-group, *n* = 67 sub-groups). We also detected 69 howler monkey groups across 30 plots and censused 117 individuals (mean ± SD = 5.3 ± 2.4 individuals per group, *n* = 22 groups). Howler monkey presence was not related to any specific vegetation type, while spider monkeys were present in areas with a higher percentage of tall forest (trees > 25 m high). Overall, spider monkeys were more prevalent than howler monkeys in our sampling sites and showed demographic characteristics similar to those in better protected areas, suggesting that the landscape features in the Uxpanapa Valley are suitable for their needs. Conversely, howler monkey presence was found to be more limited than in other regions, possibly due to the extended presence of spider monkeys.

**Supplementary Information:**

The online version contains supplementary material available at 10.1007/s10329-022-00980-8.

## Introduction

The degradation of tropical habitats has reduced the population sizes of many species to critical levels (Volis and Deng 2020). This is particularly true for non-human primates (hereafter ‘primates’), with 75% of primate species decreasing globally (Cuarón et al. 2020; Estrada et al. 2017, 2020). This is of concern, as primates represent a significant component of tropical biomass and play a critical role in maintaining tropical biodiversity, as well as processes and services supporting ecosystem function (Estrada et al. 2017). To develop effective conservation strategies for primate species, we must first have information on their distribution and demography (Campbell et al. 2016; Spaan et al. 2019), especially considering that many efforts to conserve species have been hindered by limited data on both of these parameters (Karanth et al. 2010).

Verified primate distribution and demographic data are still limited in several regions of Mexico, including the Selva Zoque Corridor. This area was highlighted as a priority location for the collection of Mexican mantled howler monkey (*Alouatta palliata mexicana*) and spider monkey (*Ateles geoffroyi*) population data by the Mexican Primate Conservation Assessment and Management Plan and the Species at Risk Conservation Program [Rodríguez-Luna et al. 2009; Secretaría de Medio Ambiente y Recursos Naturales (SEMARNAT) 2012]. These two primates are classified as Endangered on the International Union for Conservation of Nature (IUCN) Red List (Cuarón et al. 2020; Rosales-Meda et al. 2020). Hence, there is an urgent need for further demographic surveys, as the data obtained from these may be key to the future management and conservation of these primates (Dunn et al. 2014).

Forest cover, distribution and structure (and thus, food availability) are considered to be among the primary drivers of primate presence (Alcocer-Rodríguez et al. 2020; Arce-Peña et al. 2019; Galán-Acedo et al. 2019a; Gouveia et al. 2014), while bioclimatic variables such as rainfall and temperature can potentially impact primate distribution and group size (Korstjens et al. 2006; Vidal-García and Serio-Silva 2011; Williams et al. 2021). Although howler (*Alouatta* spp*.*) and spider (*Ateles* spp*.*) monkeys belong to the same family (Atelidae), their habitat requirements differ substantially (Rylands et al. 2006). Spider monkeys are usually restricted to primary rainforests and have large home ranges (Wallace 2008). They are also characterised by a high degree of fission–fusion dynamics, forming sub-groups of varying sizes throughout the day (Aureli and Schaffner 2008). In Mexico, average sub-group size in protected areas ranges from 3.5 to 7.7 individuals (Estrada et al. 2004; Ortíz-Martínez et al. 2012; Pinacho-Guendulain and Ramos-Fernández 2017) and is 4.6 individuals in fragmented sites (Solórzano-García and Rodríguez-Luna 2010). In contrast, due to behavioural and physiological adaptations (Dunn et al. 2009, 2010; Milton et al. 1980), howler monkeys can survive in smaller home ranges (Dias et al. 2014; Di Fiore et al. 2011). This enables them to live in a diverse array of habitat types, ranging from undisturbed tall evergreen forests to highly disturbed small forest fragments, woodlands and orchards (Bicca-Marques et al. 2020). In Mexico their average group size is 7.0 individuals in protected areas (Cristóbal-Azkarate et al. 2005) and ranges from 4.1 to 9.0 individuals in fragmented sites (Anzures-Dadda and Mason 2007; Puig-Lagunes et al. 2016; Solórzano-García and Rodríguez-Luna 2010).

Here, we present new distribution and demographic information for *Alouatta palliata mexicana* and *Ateles geoffroyi* from the Uxpanapa Valley, in the northern portion of the Selva Zoque Corridor, an understudied but biogeographically important region. We additionally analyse the association between primate presence and land cover types, together with altitude (as a proxy for bioclimatic variables) and human disturbance, to highlight key features for the conservation of both of these primates.

## Methods

### Study area

The Uxpanapa Valley (17°17′–17°21′N, 93°40′–94°05′W) is located in the northern part of the Selva Zoque Corridor in the state of Veracruz, Mexico, and has a total area of 6200 km^2^ (PRONATURA 2009; Ruiz-Guerra et al. 2014). The mean annual temperature of the valley is between 24 and 26 °C, with 1500–3500 mm precipitation per year (Instituto Nacional de Estadística y Geografía 2008).

Parts of the Uxpanapa Valley lie on a karst platform, making some areas highly rugged and inaccessible (J. M. Day-White, unpublished data; PRONATURA 2009). The original predominant vegetation of the region was tropical rainforest (Ruiz-Guerra et al. 2014) but severe transformation has occurred since the 1970s, with annual deforestation rates of 2.1% over the past 40 years (Gómez-Pompa 1979; Hernández-Gomez et al. 2013). The Uxpanapa Valley is one of two deforestation hotspots within the Petén-Veracruz moist forests ecoregion (Vaca et al. 2012) and agricultural practices dominate the landscape (Rodríguez-Luna et al. 2011). However, at the time of our study, large areas of relatively well-preserved rainforest remained, and the Uxpanapa Valley was classified as one of the most biodiverse areas in Mexico and the world (Arriaga et al. 2000; World Wide Fund for Nature–Fundacion Carlos Slim 2018).

### Mapping

We divided the study area into 275 plots of 5 km × 5 km to survey primates across a range of landscapes with varying degrees of deforestation and fragmentation, while maintaining a reasonable probability of finding both primate species. We only surveyed plots with a minimum forest coverage of 30% as quantified by a Landsat Thematic Mapper (2003) image for the region, with forest cover information created by PRONATURA (2009). This was considered to offer an appropriate balance of increasing the likelihood of encountering both species of primates within a 2-year time frame, while covering as much of the 6200-km^2^ area as possible. However, we acknowledge that using this criterion could potentially limit the detection of some howler monkey groups, due to their smaller home and day ranges and increased tolerance of fragmentation. We largely solved this search bias by using local knowledge of primates in the study site to find out whether any primates were present in areas with less forest cover. We randomly selected 54 of the 275 plots to survey using the ArcMap Sampling Design Tool (NOAA-ESRI add-in).

During our initial exploration of the sampling plots, we found that the vegetation classification required updating, so we created a finer-scale revision in order to provide a more accurate vegetation map. We used five SPOT-5 scenes captured in the dry season of March and April 2011 (Estación de Recepción México de la Constelación/Secretaría de Marina Armada de México 2010) to classify vegetation cover and land use in the study area. The images were projected using the Universal Transverse Mercator projection based on the World Geodetic System 1984 and mosaicked together (C. A. Muñoz-Robles, unpublished data). We established five vegetation cover/land use types for the Uxpanapa Valley: tall evergreen forest (TEF), mature secondary forest (MSF), secondary forest (SF), transformed habitat (TH), and human settlement (HS) areas. We characterised TH as rubber plantations, grassland/traditional agriculture or bare soil. We characterised SF as arboreal vegetation of ≤ 20 years of age, with a minimum height of 15 m, composed of pioneer species (e.g. *Myriocarpa longipes*, *Croton pyramidale*, *Cecropia obtusifolia*, *Heliocarpus appendiculatus*) (J. C. Lopez-Acosta et al., unpublished data). We characterised MSF as arboreal vegetation of > 20 years of age, with a height of between 15 and 25 m, containing a mix of old forest and young pioneer species. Finally, TEF had trees > 25 m in height as well as a high plant species diversity, including slow-growing and high-biomass species such as *Dialium guianense*, and *Astrocaryum mexicanum* (J. C. Lopez-Acosta et al., unpublished data). To validate the image classification, we visited 500 locations and recorded their vegetation and land cover categories, together with their coordinates, using a handheld Global Positioning System (GPS) device. Each visited location was in a homogeneous area within the vegetation or land cover type to minimise geopositional errors. The achieved overall classification accuracy was 88% (C. A. Muñoz-Robles, unpublished data). We conducted all image processing using PCI Geomatica 12 software (PCI Geomatics 2011).

We compiled bioclimatic variables for our study site using the WorldClim global climate database (http://www.worldclim.org/). We also used WorldClim altitude data, which derive from a Shuttle Radar Topography Mission 30-m resolution digital elevation model.

### Primate surveys

We limited our surveys to the dry seasons (March–June) of 2010 and 2011 because seasonal floods during the rainy season make large parts of the region inaccessible during the rest of the year. Typical distance/transect sampling was not possible in this region due to highly rugged terrain, and in some cases due to ongoing land use disputes between local communities that limited our ability to access contested areas safely.

We surveyed each plot once (for a minimum of 8 h) by listening for vocalizations and locating groups with the aid of local guides (two researchers and one local guide). We also looked for and recorded cues of primate presence (i.e. urine, latrines, fallen bitten fruits). We commenced stationary listening at strategic places between 4.30 and 5.00 a.m. (before sunrise), waiting for the howler monkeys to howl. Vocal detection was the main method of detecting howler monkeys, which produce loud calls at dawn and dusk each day, as they are much more difficult to spot than spider monkeys and relatively easy to hear at distances over 1 km (Whitehead 1995). Upon hearing a vocalization, we began our surveys and recorded the observer location (GPSMAP 60CSx; Garmin) and the direction (compass direction) from which the sound came. We then walked the area for all occurrence recording of visual and auditory cues of both primate species, covering a total of 267.2 km (in 2010) and 269.5 km (in 2011). Within each plot, the mean distance walked was 8.1 km (minimum = 4.0 km, maximum = 15.6 km, mean = 6.9 km, SD = 3.3); the distance covered was largely dependent on accessibility within the site. When we visually detected a primate group, we recorded its location (by GPS), the number of individuals and their sex and age-classes, and the vegetation type (for further ground truthing of our map).

Our primate-occurrence findings were further validated through informal interviews with villagers of the communities settled in or near the selected plots. The local authorities that granted us permission to survey in each site introduced us to people who had experience of the forest. We talked to between five and ten individuals in each of the 36 villages we visited. The informal interviews were completely voluntary and during these conversations people described the primates they saw in the area and where they were usually located. This helped us to find our local guides, and to learn whether there were monkeys in the area. Our guides and the other villagers we talked to had lived in the Uxpanapa Valley for most, if not all, of their lives, and showed extensive knowledge of the sites and the animal species in them.

We designated plots as ‘primate absent’ if both the information provided by the people we talked to and our surveys indicated there were no primates, and as ‘primates present’ if villagers said there were primates in the area and we located primates during our surveys. The information gathered in our surveys and what villagers told us agreed in all cases; we always found primates in the plots where locals indicated primate presence and vice versa. Local knowledge is not just anecdotal, but allows researchers to gather information that would otherwise be difficult to obtain using conventional methods (Ahmad et al. 2021).

We used ArcMap v. 10.1 to determine the percentage of forest types, human settlements, and transformed landscapes and annual mean temperature, mean altitude and annual mean rainfall within each plot based on our landscape classification layer and BioClim data. We also mapped the observed primate distribution within the selected plots in the Uxpanapa Valley and calculated the distance between surveyed primate groups and human settlements. We acknowledge it has been 10 years since our field study took place, and our findings on vegetation and primate presence must be interpreted within this context and with the understanding that some of the sites and/or groups we studied might have now disappeared due to human pressures. Despite our study’s limitations, the data are reliable and sufficient for further understanding of both species’ presence, distribution and demography in the region.

### Data analysis

We used generalised linear models (GLMs) with a logit link function and binomial error structure (Crawley 2007) to analyse the effect of the independent variables on (1) the presence of spider monkeys, and (2) the presence of howler monkeys (dependent variables).

The land cover predictor variables were the percentage of SF, MSF, and TEF estimated for each sampled plot. We also included as predictors the percentage of TH, the percentage of area occupied by HS, mean altitude, annual mean rainfall, annual mean temperature and the number of groups of the other primate species per plot. We selected these predictors because forest type and bioclimatic variables have been shown to influence the distribution of both *Alouatta palliata mexicana* and *Ateles geoffroyi* (Vidal-García and Serio-Silva 2011), and both are at risk from human activities that impact forests (Estrada et al. 2017). We tested for multicollinearity between predictor variables using the faraway R package (Faraway 2016) and found percentage of TH, rainfall and temperature had a large variance inflation factor (VIF). We removed these variables from our models and the remaining predictors maintained low VIFs [i.e. < 8 (Hair et al. 2010)]. We ran the GLMs using the MASS R package (Venables and Ripley 2002) and used the function dredge in the MuMIn package to select the best model based on Akaike’s information criterion (Supplementary Tables S1, S2). To determine the ability of the model to explain data variation, we compared the fit of the best model selected against a null model that included only the intercept, using a likelihood ratio test (Supplementary Table S3). We carried out all the statistical analyses in R software 3.6.3 (R Core Team 2020).

## Results

The landscape composition across all sampled plots was 33.1% TH, 28.3% SF, 23.5% TEF, 14.2% MSF, and 0.4% HS. We found at least one of the two primate species in 42 of 54 (78%) plots, with 19 (35%) having both species, 30 (55%) containing howler monkeys and 31 (57%) containing spider monkeys.

### Spider monkeys

**Fig. 1:**
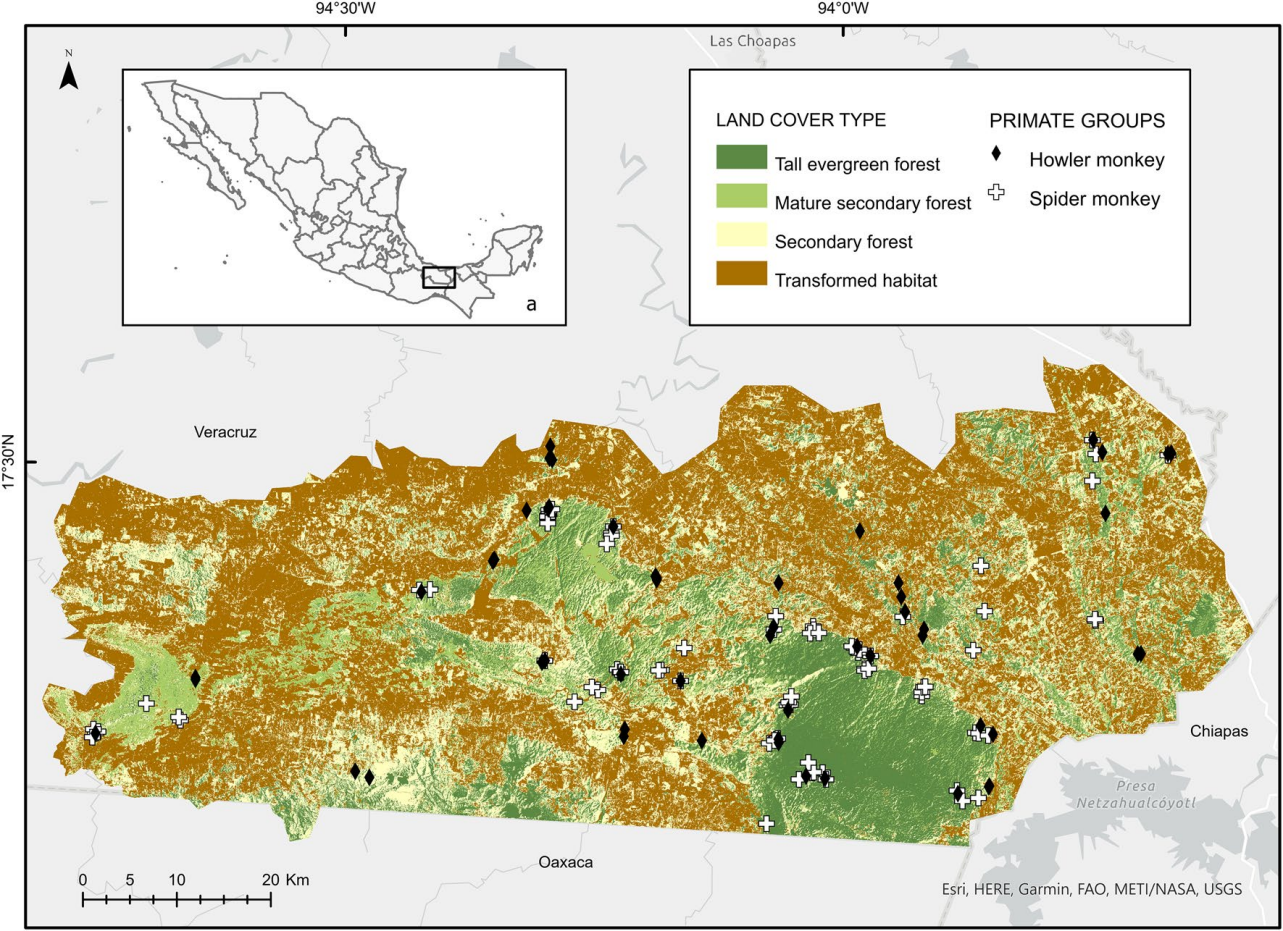
Location of the Uxpanapa Valley [**a** *black box* within the map of Mexico (*inset*)], in the State of Veracruz. *White crosses* indicate the distribution of spider monkeys,* black diamonds* the distribution of howler monkeys. Simplified land cover types of the study area are as follows: transformed habitat (*brown*), secondary forest (*light yellow*), mature secondary forest (*light green*) and tall evergreen forest (*dark green*)

We detected 86 spider monkey sub-groups in 31 plots (67 sub-groups through direct observation; eight sub-groups through direct observation, although we were unable to count the number of individuals; and 11 sub-groups detected only by vocalisations) (Fig. 1). Overall, we observed 391 spider monkeys. Sub-groups were composed of 5.9 ± 3.0 individuals (mean ± SD), of which 1.8 ± 1.4 were adult males, 2.4 ± 1.8 were adult females, 0.4 ± 0.7 were juveniles, and 0.8 ± 0.9 infants. We were not able to determine sex or age-class for 92 individuals, as on some occasions the site conditions limited our ability to view or follow them. The composition and sub-group size for spider monkeys were similar across vegetation types (Kruskal–Wallis,* H* = 3.4, *p* = 0.18; Supplementary Table S4). Most sub-groups were found in either TEF (42%) or MSF (39%), while only 19% were found in SF (Table 1). Sites inhabited by *A. geoffroyi* only had 27% of TH. The percentage of TEF was the strongest predictor of spider monkey presence (β = 0.12, SE = 0.05, *z*-value = 2.73, *p* = 0.01; Supplementary Table S5), while percentage of SF showed a negative association with presence (β = − 0.07, SE = 0.03, *z*-value = − 2.16, *p* = 0.03). The percentage of MSF, the area occupied by HS, altitude, and the presence of howler monkey groups did not significantly affect the probability of spider monkey presence (Supplementary Table S5).

**Table 1:** Group size and composition (no. of individuals; mean ± SD), and sex and age ratios of spider monkeys inhabiting three vegetation types in the Uxpanapa Valley

	Group size	Adult males	Adult females	Juveniles	Infants	U	M:F	F:I
Tall forest	5.1 ± 2.5	1.9 ± 1.8	1.6 ± 1.1	0.2 ± 0.6	0.5 ± 0.6	43	1:1.3	1:0.6
Mature secondary forest	5.8 ± 2.6	1.8 ± 1.3	2.8 ± 1.9	0.4 ± 0.6	0.7 ± 0.7	26	1:1.9	1:0.5
Secondary forest	7.8 ± 4.6	3.5 ± 2.1	2.0 ± 1.1	0.6 ± 1.1	1.8 ± 1.7	23	1:1.9	1:1

*U* Individuals whose sex could not be determined, *M:F* male-to-female ratio, *F:I* female-to-immature ratio

### Howler monkeys

We detected 69 howler monkey groups in 30 plots. We directly observed 117 individuals from 22 groups and detected another 47 groups from vocalisations (Fig. 1). Many of the areas where we detected vocalisations were located on karst walls, which were impossible to access. The observed howler monkey groups were composed of 5.3 ± 2.4 individuals (mean ± SD), of which 1.6 ± 0.7 were adult males, 2.3 ± 1.1 were adult females, 0.2 ± 0.5 were juveniles, and 1.1 ± 1.2 infants. Howler monkey group size and composition were similar across vegetation types (Kruskal–Wallis,* H* = 5.7, *p* = 0.06; Supplementary Table S4). We found 44% of the groups in MSF, 42% in TEF and 14% in SF (Table 2). Sites inhabited by howler monkeys had 37% TH. The presence of howler monkeys was not significantly related to any vegetation types, measure of disturbance or altitude, but was positively associated with the number of spider monkey groups (β = 0.41, SE = 0.18, *z*-value = 2.24, *p* = 0.03) (Supplementary Table S5).

**Table 2:** Group size and composition (no. of individuals; mean ± SD), and sex and age ratios of howler monkeys according to their presence across three vegetation types in the Uxpanapa Valley

	Group size	Adult males	Adult females	Juveniles	Infants	M:F	F:I
Tall forest	6.5 ± 2.7	1.9 ± 0.8	2.8 ± 1.1	0.3 ± 0.5	1.3 ± 1.5	1:1.8	1:0.6
Mature secondary forest	4.5 ± 1.8	1.3 ± 0.5	2.0 ± 1.1	0.3 ± 0.8	1.0 ± 0.8	1:1.8	1:0.7
Secondary forest	3.3 ± 0.6	1.3 ± 0.6	1.3 ± 0.6	0.0	0.7 ± 0.6	1:1	1:0.7

For abbreviations, see Table 1

## Discussion

Contrary to expectations, we found more plots occupied by spider than howler monkeys, despite spider monkeys being considered highly vulnerable to forest loss (Galán-Acedo et al. 2019b; Ramos-Fernandez and Wallace 2008; Spaan et al. 2020), whilst howler monkeys show adaptability to altered landscapes (Alcocer-Rodríguez et al. 2020; Bicca-Marques 2003).

Our data fill a knowledge gap on the occurrence and demographic details of both of these primates in the Selva Zoque region, even if there were more spider and howler monkey groups in the region than we were able to detect with our sampling methods. Specifically, some howler monkey groups may have been excluded due to the selection of areas with 30% forest cover, but both our observations and informal interviews with locals pointed towards howler monkeys being less common than spider monkeys. Our results also agree with those reported from nearby sites in northeastern Oaxaca, where spider monkeys also had a wider distribution than howler monkeys (Ortíz-Martínez et al. 2008).

Spider monkeys may have been more prevalent due to inter-specific competition. Competition for food and space with other primate species may limit howler monkey populations (Fedigan et al. 1998; Iwanaga and Ferrari 2002). Especially in transformed habitats, resource competition between howler monkeys and sympatric species is thought to be greater than in undisturbed landscapes (Cristóbal-Azkarate et al. 2015). While our results show that howler monkey presence was associated with spider monkey presence, this was perhaps due to them both being more prevalent in TEF and MSF, but long-term studies are needed to elucidate this issue.

Spider monkey sub-group size in our study site was relatively large compared to those found in other parts of Mexico, including Yucatan (Pinacho-Guendulain and Ramos-Fernández 2017), northeastern Oaxaca (Ortíz-Martínez et al. 2012) and the severely fragmented sites of southeastern Los Tuxtlas-one of the few sites where the same species of spider monkey and sub-species of howler monkey as in this study coexist (Solórzano-García and Rodríguez-Luna 2010). The sub-group sizes we observed are more similar to those of *A. geoffroyi* in protected reserves (Estrada et al. 2004). A possible reason for this is that, at the time of our study, the forest tracts in the Uxpanapa Valley were large enough to support relatively large spider monkey groups, and the areas surrounding them may have included arboreal elements considered as supplementary habitat (Arroyo-Rodríguez et al. 2017; Galán-Acedo et al. 2021) that provide resources for spider monkeys. For example, some sub-groups used rubber plantations to travel between forested patches (AS, personal observation).

The association found between spider monkey presence and TEF was expected, as spider monkeys heavily rely on habitats with diverse feeding resources rich in energy content (e.g. fruits), a high density and abundance of large tree species, and a large amount of continuous forest cover (Arroyo-Rodríguez et al. 2007; Calle-Rendon et al. 2019; Galán-Acedo et al. 2019b; Wallace 2008), all of which are more common in tall primary forests. Although we found some of the largest sub-groups of spider monkeys in SF, there was a negative association with spider monkey presence and this vegetation type, suggesting that the use of SF is temporary. As sub-group size fluctuates in response to food availability, with larger sub-groups formed when resources are more abundant (Asensio et al. 2008, 2009a; Rodrigues 2017; Schaffner et al. 2012), it is likely that the SF may provide seasonal resources such as fruiting trees in our study site. Nevertheless, ongoing forest loss poses a continuing threat for spider monkeys in the region (Galán-Acedo et al. 2019b), as it does in the adjacent state of Oaxaca. In some Oaxaca sites, a drop in spider monkey numbers has been attributed to the synergistic effects of habitat alteration and hunting (Ortíz-Martínez et al. 2008).

Both group size and female-to-immature (F:I) ratios are critical parameters for the assessment of trends within groups (Zucker and Clarke 2003). The spider monkey F:I ratio that we observed (Table 1) was similar to that reported for Calakmul, Mexico (1:0.7) (Estrada et al. 2004), lower than those found in other protected sites in southern Mexico (1:1.2) (Estrada et al. 2004), but higher than the F:I found in Los Tuxtlas (1:0.2) (Solórzano-García and Rodríguez-Luna 2010). Overall, our results point towards spider monkey groups appearing stable at the time of our study.

Howler monkey group sizes in our study were relatively small compared to those reported for the same sub-species in fragments with varying degrees of degradation within the northern portion of Los Tuxtlas, Mexico, in which spider monkeys are considered locally extirpated (Cristóbal-Azkarate et al. 2005). Our observed group sizes are similar to those reported for howler monkeys in less conserved areas of the southeastern Los Tuxtlas, Veracruz, where approximately 80% of the original forest cover has been transformed but they still coexist with spider monkeys (Solórzano-García and Rodríguez-Luna 2010). These smaller group sizes could be related to the prevalence of spider monkeys in the area since, where they occur in sympatry, spider monkey group size and density tend to be higher than those of howler monkeys (Aquino et al. 2015; Ortíz-Martínez et al. 2008; Solórzano-García and Rodríguez-Luna 2010). Another possible explanation for the lower-than-expected presence of howler monkeys in the region is the extremely low genetic diversity found in the groups we sampled (Dunn et al. 2014). Loss of genetic diversity can reduce the ability of populations to adapt to environmental change, as well as reduce reproductive fitness (Reed and Frankham 2003).

The howler monkey F:I ratio we observed (Table 2) is similar to that of groups of howler monkeys considered as stable (Zucker and Clarke 2003), and higher than those reported for groups in fragments of Los Tuxtlas (Cristóbal-Azkarate et al. 2005; Solórzano-García and Rodríguez-Luna 2010). This points towards the groups being stable at the time we sampled, but our results need to be considered in the context of recent reports that show negative genetic effects on *A. palliata mexicana* as a consequence of limited gene flow and inbreeding, likely due to isolation, fragmentation, and small population sizes in areas that include the Uxpanapa Valley (Solórzano-García et al. 2021).

Howler monkey presence was not significantly related to vegetation type or our measure of disturbance, which supports our expectations based on their relatively broad diet, their adaptation to leaf digestion, as well as their relatively small group sizes, home ranges and greater adaptability (Bicca-Marques 2003; Cañadas-Santiago et al. 2019; Cristóbal-Azkarate et al. 2017; Dias et al. 2014). Moreover, the areas with only *Alouatta palliata* presence contained a higher percentage of TH than those with *Ateles geoffroyi* presence, which could indicate that the howler monkeys were more exposed to human impacts. However, further studies assessing why certain factors seemingly favour the prevalence of spider monkeys but not howler monkeys in the area are needed.

Knowledge of the distribution and demography of primates constitutes a basic component for determining population viability (Campbell et al. 2016; Klass et al. 2020) and making conservation decisions. Moreover, snapshot demographic data are a highly useful source of information in conservation biology (Volis and Deng 2020), particularly if a species is under severe threat and may become locally extirpated, as may be the case for the primates in the Uxpanapa Valley region considering that some of the areas we sampled have recently been deforested (B. Solórzano-García, personal communication). We also highlight that the current IUCN Red List distribution map for *A. palliata mexicana* does not include part of the Uxpanapa Valley where we located the species, which could have implications for its conservation status.

Further emphasis should be put on preserving areas with tall evergreen forest to continue to support both of these primate species. Nevertheless, efforts should also be oriented towards encouraging landowners to maintain and continue to develop agroforestry in the areas surrounding forested sites to maintain the ‘supplementary habitat’ and connectivity found in the region (Asensio et al. 2009b). Finally, the karst walls that make this area so unique could potentially provide ‘internal’ protected areas, since they are largely inaccessible to humans but maintain vegetation elements that primates use for travelling and feeding (AS, personal observation).

There is a clear need for further assessment of unsurveyed areas to drive primate conservation and management plans, especially in sites with no protection status. Given the association between primary forest and spider monkey presence, and the current rate of forest degradation and loss within the Selva Zoque Corridor, it is urgent that those remaining areas are further assessed for their biodiversity and given the adequate legislative protection to ensure primate conservation.

## Supplementary Information

Below is the link to the electronic supplementary material.

Supplementary file1 (XLSX 16 KB)

Supplementary file2 (XLSX 17 KB)

Supplementary file3 (XLSX 11 KB)

Supplementary file4 (XLSX 11 KB)

Supplementary file5 (XLSX 13 KB)

## References

[CR1] Ahmad A, Gary D, Putra W, Sagita N, Adirahmanta SN, Miller AE (2021). Leveraging local knowledge to estimate wildlife densities in Bornean tropical rainforests. Wildl Biol.

[CR2] Alcocer-Rodríguez M, Arroyo-Rodríguez V, Galán-Acedo C, Cristóbal-Azkarate J, Asensio N, Rito KF, Hawes JE, Veá JJ, Dunn JC (2020). Evaluating extinction debt in fragmented forests: the rapid recovery of a critically endangered primate. Anim Conserv.

[CR3] Anzures-Dadda A, Manson RH (2007). Patch- and landscape-scale effects on howler monkey distribution and abundance in rainforest fragments. Anim Conserv.

[CR4] Aquino R, Zárate R, López L, García G, Charpentier E (2015). Current status and threats to *Lagothrix flavicauda* and other primates in montane forest of the región Huánuco. Primate Conserv.

[CR5] Arce-Peña NP, Arroyo-Rodríguez V, Dias PA, Franch-Pardo I, Andresen E (2019). Linking changes in landscape structure to population changes of an endangered primate. Landsc Ecol.

[CR6] Arriaga L, Espinoza JM, Aguilar C, Martínez E, Gómez L, Loa E (2000) Regiones terrestres prioritarias de México. Escala de trabajo 1:1 000 000. Comisión Nacional para el Conocimiento y uso de la Biodiversidad, México

[CR7] Arroyo-Rodríguez V, Mandujano S, Benítez-Malvido J, Cuende-Fanton C (2007). The influence of large tree density on howler monkey (*Alouatta palliata mexicana*) presence in very small rain forest fragments. Biotropica.

[CR8] Arroyo-Rodríguez V, Pérez-Elissetche GK, Ordóñez-Gómez JD, González-Zamora A, Chaves ÓM, Sánchez-López S, Chapman CA, Morales-Hernández K, Pablo-Rodríguez M, RamosFernández G (2017). Spider monkeys in human-modified landscapes: the importance of the matrix. Trop Conserv Sci.

[CR9] Asensio N, Korstjens AH, Schaffner CM, Aureli F (2008). Intragroup aggression, fission–fusion dynamics and feeding competition in spider monkeys. Behaviour.

[CR10] Asensio N, Korstjens AH, Aureli F (2009). Fissioning minimizes ranging costs in spider monkeys: a multiple-level approach. Behav Ecol Sociobiol.

[CR11] Asensio N, Arroyo-Rodríguez V, Dunn JC, Cristóbal-Azkarate J (2009). Conservation value of landscape supplementation for howler monkeys living in forest patches. Biotropica.

[CR12] Aureli F, Schaffner CM, Campbell CJ (2008). Social interactions, social relationships and the social system of spider monkeys. Spider monkeys: behavior, ecology and evolution of the genus *Ateles*.

[CR13] Bicca-Marques JC, Marsh LK (2003). How do howler monkeys cope with habitat fragmentation?. Primates in fragments: ecology and conservation.

[CR14] Bicca-Marques JC, Chaves ÓM, Hass GP (2020). Howler monkey tolerance to habitat shrinking: lifetime warranty or death sentence?. Am J Primatol.

[CR15] Calle-Rendón BR, Hilário RR, de Toledo JJ (2019). Effect of site attributes and matrix composition on Neotropical primate species richness and functional traits: a comparison among regions. Diversity.

[CR16] Campbell G, Head J, Junker J, Nekaris KAI, Wich SA, Marshall AJ (2016). Primate abundance and distribution: background concepts and methods. An introduction to primate conservation.

[CR17] Cañadas Santiago S, Dias PAD, Garau S, Coyohua Fuentes A, Chavira Ramírez DR, Canales Espinosa D, Rangel Negrín A (2019). Behavioral and physiological stress responses to local spatial disturbance and human activities by howler monkeys at Los Tuxtlas. Mexico Anim Conserv.

[CR18] Crawley M (2007). The R book.

[CR19] Cristóbal-Azkarate J, Veà JJ, Asensio N, Rodríguez-Luna E (2005). Biogeographical and floristic predictors of the presence and abundance of mantled howlers (*Alouatta palliata mexicana*) in rainforest fragments at Los Tuxtlas. Mexico Am J Primatol.

[CR20] Cristóbal-Azkarate J, Urbani B, Asensio N, Kowalewski MM, Garber PA, Cortes-Ortiz L, Urbani B, Youlatos D (2015). Interactions of howlers with other vertebrates: a review. Howler monkeys: behavior, ecology, and conservation.

[CR21] Cristóbal-Azkarate J, Dunn JC, Balcells CD, Baró JV (2017). A demographic history of a population of howler monkeys (*Alouatta palliata*) living in a fragmented landscape in Mexico. PeerJ.

[CR22] Cuarón AD, Shedden A, Rodríguez-Luna E, de Grammont PC, Link A (2020). *Alouatta palliata* ssp. *mexicana*. IUCN Red List of Threatened Species.

[CR23] Di Fiore A, Link A, Campbell CJ, Campbell CJ, Fuentes A, MacKinnon KC, Panger M, Beader SK (2011). The atelines: behavioral and socioecological diversity in a New World radiation. Primates in perspective.

[CR24] Dias PAD, Rangel-Negrín A, Coyohua-Fuentes A, Canales-Espinosa D (2014). Variation in dietary breadth among groups of black howler monkeys is not associated with the vegetation attributes of forest fragments. Am J Primatol.

[CR25] Dunn JC, Cristóbal-Azkarate J, Veà JJ (2009). Differences in diet and activity pattern between two groups of *Alouatta palliata* associated with the availability of big trees and fruit of top food taxa. Am J Primatol.

[CR26] Dunn JC, Cristóbal-Azkarate J, Veà JJ (2010). Seasonal variations in the diet and feeding effort of two groups of howlers in different sized forest fragments. Int J Primatol.

[CR27] Dunn JC, Shedden-González A, Cristóbal-Azkarate J, Cortés-Ortiz L, Rodríguez-Luna E, Knapp LA (2014). Limited genetic diversity in the critically endangered Mexican howler monkey (*Alouatta palliata mexicana*) in the Selva Zoque. Mexico Primates.

[CR28] ERMEXS, Estación de Recepción México de la Constelación SPOT/Secretaría de Marina Armada de México (2010) SPOT 5 images

[CR30] Estrada A, Luecke L, Van Belle S, Barrueta E, Meda MR (2004). Survey of black howler (*Alouatta pigra*) and spider (*Ateles geoffroyi*) monkeys in the Mayan sites of Calakmul and Yaxchilán, Mexico and Tikal. Guatem Primates.

[CR31] Estrada A, Garber PA, Rylands AB, Roos C, Fernandez-Duque E, Di Fiore A, Nekaris KA, Nijman V, Heymann EW, Lambert JE, Rovero F, Barelli C, Setchell JM, Gillespie TR, Mittermeier RA (2017). Impending extinction crisis of the world’s primates: why primates matter. Sci Adv.

[CR32] Estrada A, Garber PA, Chaudhary A (2020). Current and future trends in socio-economic, demographic and governance factors affecting global primate conservation. PeerJ.

[CR33] Faraway J (2016) Faraway: functions and datasets for books. R package version 1.0.7. https://CRAN.Rproject.org/package=faraway. Accessed September 2016

[CR34] Fedigan LM, Rose LM, Avila RM (1998). Growth of mantled howler groups in a regenerating Costa Rican dry forest. Int J Primatol.

[CR35] Galán-Acedo C, Arroyo-Rodríguez V, Andresen E, Arregoitia LV, Vega E, Peres CA (2019). The conservation value of human-modified landscapes for the world’s primates. Nat Commun.

[CR36] Galán-Acedo C, Arroyo-Rodríguez V, Cudney-Valenzuela S, Fahrig L (2019). A global assessment of primate responses to landscape structure. Biol Rev.

[CR37] Galán-Acedo C, Arroyo-Rodríguez V, Andresen E, Dias PA (2021). Regional context mediates the response of Mexican primates to landscape structure in fragmented rainforests. Biol Cons.

[CR38] PCI Geomatics (2011) Geomatica 2012. PCI Geomatics, Ontario, Canada

[CR39] Gómez-Pompa A (1979). Antecedentes de las investigaciones botánico-ecológicas en la región del Río Uxpanapa, Ver., México. Biotica.

[CR40] Gouveia SF, Villalobos F, Dobrovolski R, Beltrão Mendes R, Ferrari SF (2014). Forest structure drives global diversity of primates. J Anim Ecol.

[CR41] Hair JF, Black WC, Babin BJ, Anderson RE (2010). Multivariate data analysis.

[CR42] Hernández-Gómez I, Ellis EA, Gómez CAG (2013). Aplicación de teledetección y sistemas de información geográfica para el análisis de deforestación y deterioro de selvas tropicales en la región Uxpanapa, Veracruz GeoFocus. Revista Internacional de Ciencia y Tecnología de la Información Geográfica.

[CR43] Instituto Nacional de Estadística y Geografía (INEGI) (2008) Información referenciada geoespacialmente entegrada en un sistema. INEGI, Mexico

[CR44] Iwanaga S, Ferrari SF (2002). Geographic distribution of red howlers (*Alouatta seniculus*) in southwestern Brazilian Amazonia, with notes on *Alouatta caraya*. Int J Primatol.

[CR45] Karanth KK, Nichols JD, Hines JE (2010). Occurrence and distribution of Indian primates. Biol Cons.

[CR46] Klass K, Van Belle S, Estrada A (2020). Demographic population structure of black howler monkeys in fragmented and continuous forest in Chiapas, Mexico: implications for conservation. Am J Primatol.

[CR47] Korstjens AH, Verhoeckx IL, Dunbar RI (2006). Time as a constraint on group size in spider monkeys. Behav Ecol Sociobiol.

[CR48] Secretaría de Medio Ambiente y Recursos Naturales (SEMARNAT)/Comisión Nacional de Áreas Naturales Protegidas (CONANP) (2012) Programa de Acción para la Conservación de las Especies: Primates, Mono Araña (*Ateles geoffroyi*) y Monos Aulladores (*Alouatta palliata*, *Alouatta pigra*), In: Oropeza-Hernández P, Rendón-Hernández E (eds). México

[CR49] Milton K, Van Soest P, Robertson J (1980). Digestive efficiencies of wild howler monkeys. Physiol Zool.

[CR50] Ortíz-Martínez T, Rico-Gray V, Martínez-Meyer E (2008). Predicted and verified distributions of *Ateles geoffroyi* and *Alouatta palliata* in Oaxaca, Mexico. Primates.

[CR51] Ortíz-Martínez T, Pinacho-Guendulain B, Mayoral-Chávez P, Carranza-Rodríguez JC, Ramos-Fernández G (2012). Demografía y uso de hábitat del mono araña (*Ateles geoffroyi*) en una selva húmeda tropical del norte de Oaxaca. Mexico Therya.

[CR52] Pinacho-Guendulain B, Ramos-Fernández G (2017). Infuence of fruit availability on the fssion–fusion dynamics of spider monkeys (*Ateles geofroyi*). Int J Primatol.

[CR53] PRONATURA (2009) Evaluación del estado de conservación de los ecosistemas forestales de la región denominada Uxpanapa. PRONATURA, Mexico, pp 31–37

[CR54] Puig-Lagunes ÁA, Canales-Espinosa D, Rangel-Negrín A, Dias PAD (2016). The influence of spatial attributes on fragment occupancy and population structure in the Mexican mantled howler (*Alouatta palliata mexicana*). Int J Primatol.

[CR55] R Core Team (2020) R: a language and environment for statistical computing. R Foundation for Statistical Computing, Vienna, Austria. URL http://www.R-project.org/ Accessed Sept 2021

[CR56] Ramos-Fernandez G, Wallace RB, Campbell CJ (2008). Spider monkey conservation in the twenty-first century: recognizing risks and opportunities. Spider monkeys: the biology, behavior and ecology of the genus *Ateles*.

[CR57] Reed DH, Frankham R (2003). Correlation between fitness and genetic diversity. Conserv Biol.

[CR58] Rodrigues MA (2017). Female spider monkeys (*Ateles geoffroyi*) cope with anthropogenic disturbance through fission–fusion dynamics. Int J Primatol.

[CR59] Rodríguez-Luna E, Solórzano-García B, Shedden-González A, Rangel-Negrín A, Dias PA, Cristobal-Azkarate J, Cortés-Ortíz L, Dunn JC, Domingo-Balcells C, Sánchez S, Vea-Baro J, Cornejo L (2009) Taller de conservación, análisis y manejo planificado para los primates mexicanos (CAMP), 2006. Universidad Veracruzana/CBSG-México

[CR60] Rodríguez-Luna E, Gómez-Pompa A, López JC, Velázquez-Rosas N, Aguilar Y, Vázquez M (2011) Atlas de los espacios naturales protegidos de Veracruz. Secretaria de Educación, Gobierno del Estado de Veracruz, Mexico

[CR61] Rosales-Meda M, Cortes-Ortíz L, Canales Espinosa D, Marsh LK, Rylands AB, Mittermeier RA (2020) *Ateles geoffroyi* ssp. *vellerosus*. The IUCN Red List of Threatened Species. 10.2305/IUCN.UK.2020-3.RLTS.T160872795A17979441.en

[CR62] Ruiz-Guerra B, Rosas NV, López-Acosta JC (2014). Plant diversity in live fences and pastures, two examples from the Mexican humid tropics. Env Manag.

[CR63] Rylands AB, Groves CP, Mittermeier RA, Cortez-Ortiz L, Hines JJ, Estrada A, Garber PA, Pavelka M, Luecke L (2006). Taxonomy and distributions of Mesoamerican primates. New perspectives in the study of Mesoamerican primates: distribution, ecology, behaviour, and conservation.

[CR64] Schaffner CM, Rebecchini L, Ramos-Fernández G (2012). Spider monkeys (*Ateles geoffroyi yucatenensis*) cope with the negative consequences of hurricanes through changes in diet, activity budget, and fission–fusion dynamics. Int J Primatol.

[CR65] Solórzano-García B, Luna ER (2010). Cambios demográficos en poblaciones de primates de la region Sur de Los Tuxtlas, México: análisis longitudinal 1985–2008. Neotrop Primates.

[CR66] Solórzano-García B, Zubillaga D, Piñero D, Vázquez-Domínguez E (2021). Conservation implications of living in forest remnants: inbreeding and genetic structure of the northernmost mantled howler monkeys. Biotropica.

[CR67] Spaan D, Burke C, McAree O, Aureli F, Rangel-Rivera CE, Hutschenreiter A, Longmore SN, McWhirter PR, Wich SA (2019). Thermal infrared imaging from drones offers a major advance for spider monkey surveys. Drones.

[CR68] Spaan D, Ramos-Fernández G, Bonilla-Moheno M, Schaffner CM, Morales-Mávil JE, Slater K, Aureli F (2020). Anthropogenic habitat disturbance and food availability affect the abundance of an endangered primate: a regional approach. Mamm Biol.

[CR69] Vaca RA, Golicher DJ, Cayuela L, Hewson J, Steininger M (2012) Evidence of incipient forest transition in southern Mexico. Plos One 7(8): e42309 10.1371/journal.pone.0042309 PMC3414527 22905123

[CR70] Venables WN, Ripley BD (2002) Modern applied statistics with S, fourth edition. Springer, New York. ISBN 0–387–95457–0 http://www.stats.ox.ac.uk/pub/MASS4/ Accessed Sept 2020

[CR71] Vidal-García F, Serio-Silva JC (2011). Potential distribution of Mexican primates: modeling the ecological niche with the maximum entropy algorithm. Primates.

[CR72] Volis S, Deng T (2020). Importance of a single population demographic census as a first step of threatened species conservation planning. Biodivers Conserv.

[CR73] Wallace RB, Campbell CJ (2008). Factors influencing spider monkey habitat use and ranging patterns. Spider monkeys: behavior, ecology and evolution of the genus *Ateles*.

[CR74] Whitehead JM (1995). Vox Alouattinae—a preliminary survey of the acoustic characteristics of long distance calls of howling monkeys. Int J Primatol.

[CR75] Williams KA, Slater HD, Gillingham P, Korstjens AH (2021). Environmental factors are stronger predictors of primate species’ distributions than basic biological traits. Int J Primatol.

[CR76] World Wide Fund for Nature–Fundacion Carlos Slim (2018) Selva Zoque. https://fundacioncarlosslim.org/conoce-trabajo-la-alianza-wwf-fundacion-carlos-slim-en-selva-zoque/ Accessed 19 Jun 2021

[CR77] Zucker EL, Clark MR (2003). Longitudinal assessment of immature-to-adult ratios in two groups of Costa Rican *Alouatta palliata*. Int J Primatol.

